# Audit Report on Leave and Observation Documentation in Admission Plans for Out-of-Hours Admissions

**DOI:** 10.1192/bjo.2026.11573

**Published:** 2026-06-30

**Authors:** Topias Hokkanen, Rizwan Ahmad, Rashed Alhadi

**Affiliations:** NHS Tayside, Dundee, United Kingdom

## Abstract

**Aims:**

Assessing risk is a fundamental skill in psychiatry, particularly for Core Trainees who are developing their skills during Out-of-Hours (OOH) shifts. However, the time pressure in these shifts often leads doctors to quickly document their admission notes without much thought for risk management. Failing to record Mental Health Act (MHA) status, leave permissions, or observation levels may create significant safety gaps and falls short of the standards set by the Mental Health (Care and Treatment) (Scotland) Act 2003. We retrospectively collected data that supported this notion and posted simple, structured posters to guide trainees in robust risk documentation. The goal was to encourage active thought processing that aligns with local risk protocols, ensuring that documentation isn't just a box-ticking exercise but a helpful guide for nursing staff. This helps empowers nurses to use their discretion in line with the admitting doctors opinion and adjust care safely as a patient's risk fluctuates on the ward.

**Methods:**

We conducted a retrospective audit (Cycle 1, n=52) of OOH admission notes which showed only 15.4% of notes documented observation levels and 13.5% failed to mention leave status entirely. In response, we placed posters in key resident doctor work areas to act as a visual “nudge,” explicitly prompting doctors to define legal status and safety monitoring plans. A re-audit (Cycle 2, n=29) was completed one month later to see if these visual cues changed behaviour.

**Results:**

The second cycle showed a distinct improvement in how risk was recorded. Documentation of observation levels rose from 15.4% to 55.2%. Documentation regarding leave status also improved significantly, reaching 65.5% vs 1.9%. MHA status documentation remained roughly consistent (82.7% vs 75.9%). The rise in documenting recommended observation and leave status suggests that trainees are no longer relying on implied safety levels and are instead engaging in active risk assessment when there is a clear reminder to prompt them.

**Conclusion:**

The marked increase in recording observation and leave decisions suggests that visual prompts are an effective way to remind trainees to pause and reflect on their risk assessment and how this applies to the patient’s admission journey. The posters helped convert risk assessment from an administrative afterthought into a conscious and adaptable management strategy. This simple intervention not only improves compliance but fosters a culture where active risk management is embedded into the admission process, ultimately ensuring safer handovers and better support for the nursing team.

